# Supplementary material for: Delayed correlation between the incidence rate of indigenous murine typhus in humans and the seropositive rate of Rickettsia typhi infection in small mammals in Taiwan from 2007–2019
Source: PLoS Negl Trop Dis. 2022 Apr 25;16(4):e0010394. doi: 10.1371/journal.pntd.0010394 (PMC9071160; doi:10.1371/journal.pntd.0010394)
Supplement: S1 Table — (DOCX) [file pntd.0010394.s001.docx]

**S1 Table.** Seropositive rate of anti-*R. typhi* responses in small mammals and incidence rate (per 100,000 person-years) of indigenous murine typhus for each month from 2007–2019.

| Year | Rate | Jan | Feb | Mar | Apr | May | Jun | Jul | Aug | Sep | Oct | Nov | Dec |
| --- | --- | --- | --- | --- | --- | --- | --- | --- | --- | --- | --- | --- | --- |
| 2007 | seropositive rate | 15.22%  (7/46) | 21.43%  (9/42) | 16.28%  (7/43) | 37.14%  (13/35) | 23.08%  (12/52) | 12.5%  (6/48) | 2.86%  (1/35) | 7.69%  (4/52) | 17.86%  (5/28) | 4.55%  (2/44) | 10%  (3/30) | 0%  (0/29) |
|  | incidence rate  (per 100,000 person-years) | 0.21 | 0.10 | 0.26 | 0.21 | 0.31 | 0.47 | 0.26 | 0 | 0.16 | 0.05 | 0.16 | 0.10 |
| 2008 | seropositive rate | 4.55%  (3/66) | 7.69%  (2/26) | 15.38%  (4/26) | 17.39%  (8/46) | 16.22%  (6/37) | 21.43%  (9/42) | 9.64%  (8/83) | 8.06%  (5/62) | 15.79%  (9/57) | 5.26%  (3/57) | 11.43%  (4/35) | 0%  (0/34) |
|  | incidence rate  (per 100,000 person-years) | 0.05 | 0.05 | 0.05 | 0.05 | 0.26 | 0.10 | 0.42 | 0.05 | 0.05 | 0.16 | 0.10 | 0.05 |
| 2009 | seropositive rate | 2.63%  (1/38) | 1.92%  (1/52) | 2.22%  (1/45) | 11.11%  (4/36) | 8.51%  (4/47) | 11.32%  (6/53) | 18.18%  (8/44) | 0%  (0/36) | 8.33%  (5/60) | 7.14%  (3/42) | 3.45%  (2/58) | 18%  (9/50) |
|  | incidence rate  (per 100,000 person-years) | 0.10 | 0.16 | 0.26 | 0.10 | 0.16 | 0.10 | 0.36 | 0.26 | 0.21 | 0.10 | 0.05 | 0.05 |
| 2010 | seropositive rate | 18.92%  (7/37) | 5.77%  (3/52) | 8.7%  (4/46) | 9.3%  (4/43) | 19.23%  (10/52) | 5.88%  (3/51) | 5.77%  (3/52) | 11.59%  (8/69) | 4.29%  (3/70) | 1.41%  (1/71) | 0%  (0/75) | 7.94%  (5/63) |
|  | incidence rate  (per 100,000 person-years) | 0.10 | 0 | 0.26 | 0 | 0.41 | 0.10 | 0.41 | 0.21 | 0.21 | 0.10 | 0.10 | 0.05 |
| 2011 | seropositive rate | 13.43%  (9/67) | 7.69%  (3/39) | 14.89%  (7/47) | 7.34%  (8/109) | 15.15%  (15/99) | 3.17%  (2/63) | 11.25%  (9/80) | 3.85%  (2/52) | 10.32%  (13/126) | 6.63%  (12/181) | 6.94%  (5/72) | 6.25%  (3/48) |
|  | incidence rate  (per 100,000 person-years) | 0.16 | 0.05 | 0.05 | 0.10 | 0.21 | 0.36 | 0 | 0.05 | 0.16 | 0.05 | 0.10 | 0 |
| 2012 | seropositive rate | 2.22%  (1/45) | 1.85%  (1/54) | 4.49%  (4/89) | 5.47%  (7/128) | 12.64%  (11/87) | 0%  (0/57) | 11.11%  (9/81) | 6.94%  (5/72) | 4.84%  (3/62) | 2.94%  (2/68) | 8.82%  (3/34) | 10.53%  (4/38) |
|  | incidence rate  (per 100,000 person-years) | 0.10 | 0.05 | 0 | 0.36 | 0.15 | 0.26 | 0.05 | 0.05 | 0.21 | 0.21 | 0.15 | 0.05 |
| 2013 | seropositive rate | 2.22%  (1/45) | 3.7%  (1/27) | 14.89%  (7/47) | 21.43%  (9/42) | 7.79%  (6/77) | 12.28%  (7/57) | 8.93%  (5/56) | 12.9%  (8/62) | 9.8%  (5/51) | 14.04%  (8/57) | 6.41%  (5/78) | 11.11%  (5/45) |
|  | incidence rate  (per 100,000 person-years) | 0.10 | 0 | 0.10 | 0 | 0.26 | 0.26 | 0.26 | 0.15 | 0.05 | 0 | 0 | 0.15 |
| 2014 | seropositive rate | 5.88%  (4/68) | 3.7%  (2/54) | 8.7%  (4/46) | 8.47%  (5/59) | 13.11%  (8/61) | 9.52%  (4/42) | 17.5%  (7/40) | 9.23%  (6/65) | 7.27%  (4/55) | 3.28%  (2/61) | 1.64%  (1/61) | 6.52%  (3/46) |
|  | incidence rate  (per 100,000 person-years) | 0 | 0.05 | 0.05 | 0.10 | 0.26 | 0 | 0.15 | 0.10 | 0.10 | 0.05 | 0.10 | 0 |
| 2015 | seropositive rate | - | - | 7.14%  (4/56) | - | - | 19.15%  (9/47) | 0%  (0/4) | - | 13.73%  (7/51) | - | 4.55%  (3/66) | - |
|  | incidence rate  (per 100,000 person-years) | 0 | 0 | 0.05 | 0.26 | 0.15 | 0.51 | 0.15 | 0.26 | 0.10 | 0.10 | 0.05 | 0.10 |
| 2016 | seropositive rate | 0%  (0/4) | 0%  (0/2) | 8.7%  (6/69) | 0%  (0/2) | 0%  (0/1) | 8.06%  (5/62) | - | - | 4.35%  (3/69) | 0%  (0/1) | 6.67%  (6/90) | 14.29%  (1/7) |
|  | incidence rate  (per 100,000 person-years) | 0.05 | 0 | 0 | 0 | 0.20 | 0.15 | 0.05 | 0.10 | 0 | 0 | 0.05 | 0.05 |
| 2017 | seropositive rate | 0%  (0/2) | 0%  (0/4) | 2.94%  (2/68) | 0%  (0/4) | 0%  (0/2) | 0%  (0/72) | - | 0%  (0/1) | 0%  (0/51) | 0%  (0/3) | 3.8%  (3/79) | 0%  (0/1) |
|  | incidence rate  (per 100,000 person-years) | 0 | 0.15 | 0.10 | 0.20 | 0.46 | 0.25 | 0.15 | 0.31 | 0 | 0.15 | 0 | 0.10 |
| 2018 | seropositive rate | 0%  (0/7) | 0%  (0/2) | 4.65%  (2/43) | 0%  (0/2) | 0%  (0/3) | 9.26%  (5/54) | 12.5%  (1/8) | 0%  (0/2) | 7.06%  (6/85) | 0%  (0/6) | 5.26%  (4/76) | 14.29%  (1/7) |
|  | incidence rate  (per 100,000 person-years) | 0.10 | 0.05 | 0.05 | 0 | 0.31 | 0.36 | 0.05 | 0 | 0.10 | 0.05 | 0 | 0 |
| 2019 | seropositive rate | 0%  (0/2) | - | 6.15%  (4/65) | - | - | 8.77%  (5/57) | 0%  (0/1) | - | 1.41%  (1/71) | - | 2.25%  (2/89) | 0%  (0/1) |
|  | incidence rate  (per 100,000 person-years) | 0.05 | 0 | 0.05 | 0.05 | 0.05 | 0.36 | 0.15 | 0.20 | 0.25 | 0.10 | 0.10 | 0 |
