# Supplementary material for: Delayed correlation between the incidence rate of indigenous murine typhus in humans and the seropositive rate of Rickettsia typhi infection in small mammals in Taiwan from 2007–2019
Source: PLoS Negl Trop Dis. 2022 Apr 25;16(4):e0010394. doi: 10.1371/journal.pntd.0010394 (PMC9071160; doi:10.1371/journal.pntd.0010394)
Supplement: S2 Table — (DOCX) [file pntd.0010394.s002.docx]

**S2 Table.** Seropositive rate of anti-*R. typhi* responses in different small mammals for each study port from 2007–2019.

| **Port** | **2007** | **2008** | **2009** | **2010** | **2011** | **2012** | **2013** | **2014** | **2015** | **2016** | **2017** | **2018** | **2019** | **Total (2007-2019)** |
| --- | --- | --- | --- | --- | --- | --- | --- | --- | --- | --- | --- | --- | --- | --- |
| **(A) *Rattus norvegicus*** | | | | | | | | | | | | | | |
| Anping int'l seaport | － | － | － | － | － | － | － | － | － | － | 0 (0/2) | 0 (0/4) | 16.67% (1/6) | 8.33% (1/12) |
| Badouzi fishing port | － | － | 0 (0/2) | － | － | － | － | － | － | － | － | － | － | 0 (0/2) |
| Fuao int'l seaport | 0 (0/25) | 0 (0/20) | 0 (0/19) | 0 (0/8) | 0 (0/1) | 0 (0/6) | 0 (0/2) | 0 (0/9) | － | － | － | － | － | 0 (0/90) |
| Hoping int'l seaport | 0 (0/2) | 0 (0/2) | 0 (0/2) | 0 (0/5) | 0 (0/1) | － | － | － | － | － | － | － | 0 (0/1) | 0 (0/13) |
| Hsinchu/Haishan fishing ports | － | 0 (0/32) | － | － | － | － | － | － | － | － | － | － | － | 0 (0/32) |
| Hualien int'l airport | － | － | － | － | － | － | － | － | － | － | 0 (0/3) | － | 0 (0/1) | 0 (0/4) |
| Hualien int'l seaport | 0 (0/10) | 6.67% (1/15) | 7.14% (1/14) | 0 (0/9) | 0 (0/4) | 0 (0/2) | 0 (0/5) | － | － | 0 (0/1) | 0 (0/5) | 0 (0/1) | 0 (0/1) | 2.99% (2/67) |
| Kaohsiung int'l airport | 71.43% (10/14) | 77.78% (7/9) | 0 (0/3) | 0 (0/5) | 0 (0/5) | 0 (0/1) | 0 (0/2) | 0 (0/2) | 0 (0/1) | 0 (0/3) | 0 (0/1) | 0 (0/1) | 0 (0/3) | 32.08% (17/53) |
| Kaohsiung int'l seaport | 50.00% (34/68) | 52.87% (46/87) | 42.37% (25/59) | 47.06% (32/68) | 26.09% (66/253) | 28.05% (46/164) | 42.62% (52/122) | 47.25% (43/91) | 59.09% (13/22) | 41.67% (15/36) | 7.69% (2/26) | 53.85% (14/26) | 63.64% (7/11) | 38.24% (395/1,033) |
| Keelung int'l seaport | 0 (0/31) | 0 (0/37) | 4.26% (2/47) | 0 (0/47) | 0 (0/35) | 0 (0/18) | 0 (0/30) | 0 (0/20) | 0 (0/10) | 0 (0/9) | 0 (0/5) | 0 (0/18) | 0 (0/13) | 0.63% (2/320) |
| Liaoluo int'l seaport | 0 (0/8) | 0 (0/28) | 0 (0/12) | 3.23% (1/31) | 5.41% (2/37) | 0 (0/13) | 0 (0/2) | 0 (0/4) | 0 (0/5) | 0 (0/3) | － | － | 0 (0/1) | 2.08% (3/144) |
| Magong int'l seaport | － | － | 20.00% (1/5) | 0 (0/3) | 0 (0/2) | 0 (0/4) | 0 (0/6) | 0 (0/2) | － | － | － | － | － | 4.55% (1/22) |
| Mailiao int'l seaport | 6.45% (2/31) | 0 (0/24) | 40.00% (4/10) | 0 (0/18) | 0 (0/13) | 0 (0/5) | 0 (0/9) | 0 (0/12) | 0 (0/6) | 0 (0/7) | 0 (0/12) | 0 (0/6) | 9.09% (1/11) | 4.27% (7/164) |
| Other nameless/small ports in Matsu | － | － | 0 (0/5) | 0 (0/3) | － | － | － | － | － | － | － | － | － | 0 (0/8) |
| Other nameless/small ports in Taichung | － | － | － | － | － | － | 0 (0/2) | － | － | － | － | － | － | 0 (0/2) |
| Shuitou int'l seaport | － | 0 (0/10) | 0 (0/12) | 0 (0/10) | 0 (0/11) | 0 (0/5) | 18.18% (2/11) | 0 (0/7) | － | 0 (0/1) | － | － | 0 (0/2) | 2.9% (2/69) |
| Suao int'l seaport | 4.26% (2/47) | 0 (0/63) | 2.70% (1/37) | 0 (0/25) | 0 (0/49) | 0 (0/27) | 0 (0/21) | 0 (0/36) | 0 (0/10) | 0 (0/14) | 0 (0/6) | 0 (0/4) | 0 (0/4) | 0.88% (3/340) |
| Taichung int'l airport | － | － | － | － | － | 0 (0/1) | 0 (0/1) | 0 (0/13) | 0 (0/4) | － | － | 0 (0/1) | 0 (0/2) | 0 (0/22) |
| Taichung int'l seaport | 40.91% (9/22) | 31.25% (5/16) | 25.00% (2/8) | 41.67% (15/36) | 35.48% (11/31) | 23.08% (3/13) | 29.41% (5/17) | 24.00% (6/25) | 58.33% (7/12) | 16.67% (4/24) | 25.00% (3/12) | 40.00% (4/10) | 10.00% (3/30) | 30.08% (77/256) |
| Tainan int'l airport | － | － | － | － | － | － | － | － | － | － | 0 (0/1) | 0 (0/1) | － | 0 (0/2) |
| Taipei int'l airport | － | － | 0 (0/2) | － | 50.00% (3/6) | 0 (0/3) | 0 (0/1) | － | － | － | 0 (0/1) | 0 (0/1) | 0 (0/1) | 20.00% (3/15) |
| Taipei int'l seaport | － | － | － | － | 0 (0/3) | 0 (0/2) | － | － | － | 0 (0/1) | 0 (0/2) | 0 (0/2) | 0 (0/1) | 0 (0/11) |
| Taoyuan int'l airport | － | 0 (0/18) | 0 (0/31) | 0 (0/48) | 0 (0/62) | 0.73% (1/137) | 0 (0/38) | 0 (0/37) | 0 (0/29) | 0 (0/41) | 0 (0/19) | 0 (0/34) | 0 (0/2) | 0.20% (1/496) |
| Xinhu/Fuguodun/Lotsuo fishing ports | － | － | － | 0 (0/1) | － | － | － | － | － | － | － | － | － | 0 (0/1) |
| Yenpu/Fangliao/Shuidiliao fishing ports | － | 0 (0/6) | － | － | － | － | － | － | － | － | － | － | － | 0 (0/6) |
| Subtotal | 22.09% (57/258) | 16.08% (59/367) | 13.43% (36/268) | 15.00% (48/320) | 15.98% (82/513) | 12.47% (50/401) | 21.93% (59/269) | 18.99% (49/258) | 20.20% (20/99) | 13.57% (19/140) | 5.26% (5/95) | 16.51% (18/109) | 13.79% (12/87) | 16.14% (514/3,184) |
| **(B) *Suncus murinus*** | | | | | | | | | | | | | | |
| Anping int'l seaport | － | － | － | － | － | － | － | － | － | 0 (0/8) | 0 (0/3) | 0 (0/4) | 0 (0/1) | 0 (0/16) |
| Badouzi fishing port | － | － | 0 (0/3) | － | － | － | － | － | － | － | － | － | － | 0 (0/3) |
| Baisha int'l seaport | － | － | － | － | － | － | － | － | － | 0 (0/5) | 0 (0/2) | 0 (0/6) | 0 (0/5) | 0 (0/18) |
| Fuao int'l seaport | 0 (0/16) | 0 (0/15) | 0 (0/14) | 0 (0/3) | 0 (0/5) | 0 (0/1) | 0 (0/12) | 0 (0/25) | 0 (0/5) | 0 (0/9) | 0 (0/7) | 0 (0/8) | 0 (0/9) | 0 (0/129) |
| Hoping int'l seaport | 0 (0/2) | － | － | 0 (0/3) | － | － | － | － | － | － | － | － | － | 0 (0/5) |
| Hsinchu/Haishan fishing ports | － | 0 (0/2) | － | － | － | － | － | － | － | － | － | － | － | 0 (0/2) |
| Hualien int'l airport | － | － | － | － | － | － | － | － | － | 0 (0/7) | 0 (0/6) | 0 (0/2) | 0 (0/7) | 0 (0/22) |
| Hualien int'l seaport | 0 (0/7) | 0 (0/4) | 0 (0/5) | 0 (0/24) | 0 (0/4) | 0 (0/1) | 0 (0/5) | 0 (0/3) | － | 0 (0/2) | 0 (0/8) | 0 (0/7) | 0 (0/30) | 0 (0/100) |
| Kaohsiung int'l airport | － | 0 (0/2) | 0 (0/2) | 0 (0/39) | 0 (0/53) | 0 (0/25) | 0 (0/35) | 0 (0/19) | 0 (0/8) | 0 (0/13) | 0 (0/13) | 0 (0/1) | 0 (0/6) | 0 (0/216) |
| Kaohsiung int'l seaport | 0 (0/11) | 0 (0/14) | 0 (0/16) | 0 (0/16) | 0 (0/68) | 0 (0/101) | 7.69% (3/39) | 0 (0/30) | 0 (0/15) | 0 (0/12) | 0 (0/16) | 0 (0/15) | 0 (0/12) | 0.82% (3/365) |
| Keelung int'l seaport | 0 (0/4) | 0 (0/1) | 0 (0/10) | － | － | － | 0 (0/1) | 0 (0/1) | 0 (0/2) | 0 (0/2) | － | 0 (0/5) | 0 (0/1) | (0/27) |
| Liaoluo int'l seaport | 0 (0/14) | 0 (0/4) | 0 (0/11) | 0 (0/15) | 0 (0/19) | 0 (0/17) | 0 (0/36) | 0 (0/38) | 0 (0/13) | 0 (0/21) | 0 (0/17) | 0 (0/28) | 0 (0/13) | 0 (0/246) |
| Magong int'l seaport | － | － | － | 0 (0/4) | 0 (0/5) | 0 (0/7) | 0 (0/21) | 0 (0/25) | 0 (0/7) | 0 (0/6) | 0 (0/7) | 0 (0/5) | 0 (0/5) | 0 (0/92) |
| Mailiao int'l seaport | 10.00% (2/20) | 0 (0/2) | 18.18% (2/11) | 0 (0/12) | 0 (0/17) | 0 (0/17) | 0 (0/28) | 0 (0/19) | 0 (0/3) | 0 (0/3) | 0 (0/4) | 0 (0/10) | 0 (0/3) | 2.68% (4/149) |
| Other nameless/small ports in Matsu | － | － | 0 (0/6) | 0 (0/10) | － | － | － | － | － | － | － | － | － | 0 (0/13) |
| Shuitou int'l seaport | 0 (0/18) | 0 (0/17) | 0 (0/38) | 0 (0/44) | 0 (0/39) | 0 (0/26) | 0 (0/26) | 0 (0/40) | 0 (0/20) | 0 (0/16) | 0 (0/18) | 0 (0/33) | 0 (0/26) | 0 (0/361) |
| Taichung int'l airport | － | － | － | － | 10.00% (1/10) | 0 (0/1) | 0 (0/5) | 0 (0/2) | 0 (0/3) | 0 (0/8) | 0 (0/2) | － | － | 3.23% (1/31) |
| Taichung int'l seaport | 0 (0/8) | 0 (0/13) | 0 (0/7) | 0 (0/6) | 0 (0/15) | 0 (0/3) | 0 (0/16) | 0 (0/14) | 0 (0/3) | 0 (0/4) | 0 (0/8) | 0 (0/5) | 0 (0/16) | 0 (0/118) |
| Tainan int'l airport | － | － | － | － | － | － | － | － | － | － | 0 (0/6) | 0 (0/7) | 0 (0/5) | 0 (0/18) |
| Taipei int'l airport | － | － | － | － | 0 (0/14) | 0 (0/11) | 0 (0/7) | 0 (0/3) | － | 0 (0/3) | 0 (0/2) | 0 (0/2) | 0 (0/5) | 0 (0/47) |
| Taipei int'l seaport | － | － | 0 (0/5) | 0 (0/7) | － | 0 (0/9) | － | 0 (0/11) | 0 (0/1) | 0 (0/2) | 0 (0/3) | 0 (0/4) | 0 (0/4) | 0 (0/46) |
| Taoyuan int'l airport | － | － | 0 (0/2) | 0 (0/3) | 1.25% (1/80) | 0 (0/109) | 0 (0/30) | 0 (0/25) | 0 (0/4) | 0 (0/4) | 0 (0/8) | 0 (0/3) | 0 (0/11) | 0.36% (1/279) |
| Xinhu/Fuguodun/Lotsuo fishing ports | － | － | － | 0 (0/23) | － | － | － | － | － | － | － | － | － | 0 (0/23) |
| Yenpu/Fangliao/Shuidiliao fishing ports | － | 0 (0/3) | － | － | － | － | － | － | － | － | － | － | － | 0 (0/3) |
| Subtotal | 0 (0/13) | 0 (0/25) | 3.45% (3/87) | 2.00% (2/100) | 3.60% (4/111) | 0 (0/62) | 2.33% (2/86) | 0 (0/81) | 0 (0/19) | 0 (0/37) | 0 (0/50) | 0 (0/28) | 0 (0/20) | 1.53% (11/719) |
| **(C) *Rattus losea*** | | | | | | | | | | | | | | |
| Baisha int'l seaport | － | － | － | － | － | － | － | － | － | 0 (0/1) | － | － | － | 0 (0/1) |
| Fuao int'l seaport | － | 0 (0/2) | － | － | － | － | 0 (0/2) | 0 (0/2) | － | 0 (0/4) | 0 (0/1) | 0 (0/1) | － | 0 (0/12) |
| Hoping int'l seaport | － | － | － | － | 0 (0/1) | 0 (0/1) | 0 (0/6) | － | 0 (0/3) | 0 (0/3) | 0 (0/4) | 0 (0/1) | 0 (0/1) | 0 (0/20) |
| Hualien int'l airport | － | － | － | － | － | － | － | － | － | 0 (0/1) | － | － | － | 0 (0/1) |
| Hualien int'l seaport | － | － | － | 0 (0/5) | － | － | 2.94% (1/34) | 0 (0/27) | 0 (0/9) | 0 (0/17) | 0 (0/15) | 0 (0/11) | 0 (0/7) | 0.80% (1/125) |
| Keelung int'l seaport | － | － | － | － | － | － | － | － | － | － | － | 0 (0/4) | － | 0 (0/4) |
| Liaoluo int'l seaport | － | － | 7.69% (1/13) | 0 (0/5) | 0 (0/9) | 0 (0/6) | 0 (0/4) | 0 (0/7) | － | 0 (0/1) | 0 (0/2) | － | － | 2.13% (1/47) |
| Shuitou int'l seaport | － | － | 3.77% (2/53) | 0 (0/37) | 0 (0/62) | 0 (0/42) | 0 (0/16) | 0 (0/7) | 0 (0/7) | 0 (0/6) | 0 (0/15) | 0 (0/2) | 0 (0/2) | 0.80% (2/249) |
| Suao int'l seaport | － | － | － | － | － | － | － | － | － | － | 0 (0/1) | 0 (0/3) | 0 (0/1) | 0 (0/5) |
| Taichung int'l seaport | － | 0 (0/2) | 0 (0/1) | 50.00% (1/2) | 37.50% (3/8) | 0 (0/2) | 14.29% (1/7) | 0 (0/5) | － | 0 (0/2) | 0 (0/1) | － | － | 16.67% (5/30) |
| Tainan int'l airport | － | － | － | － | － | － | － | － | － | － | － | 0 (0/1) | － | 0 (0/1) |
| Taipei int'l airport | － | － | － | 0 (0/4) | 0 (0/1) | － | － | － | － | － | － | － | 0 (0/2) | 0 (0/7) |
| Taipei int'l seaport | － | － | 0 (0/5) | 0 (0/28) | 4.00% (1/25) | 0 (0/2) | 0 (0/14) | 0 (0/3) | － | 0 (0/1) | 0 (0/2) | － | － | 1.25% (1/80) |
| Taoyuan int'l airport | 0 (0/13) | 0 (0/19) | 0 (0/15) | 7.14% (1/14) | 0 (0/5) | 0 (0/9) | 0 (0/3) | 0 (0/30) | － | 0 (0/1) | 0 (0/9) | 0 (0/5) | 0 (0/7) | 0.77% (1/130) |
| Xinhu/Fuguodun/Lotsuo fishing ports | － | 0 (0/2) | － | 0 (0/5) | － | － | － | － | － | － | － | － | － | 0 (0/7) |
| Subtotal | 2.00% (2/100) | 0 (0/77) | 1.54% (2/130) | 0 (0/209) | 0.61% (2/329) | 0 (0/328) | 1.15% (3/261) | 0 (0/255) | 0 (0/84) | 0 (0/125) | 0 (0/130) | 0 (0/145) | 0 (0/159) | 0.39% (9/2,332) |
| **(D) Other small mammals** | | | | | | | | | | | | | | |
| Fuao int'l seaport | － | － | 0 (0/2) | 0 (0/1) | 0 (0/3) | 0 (0/4) | 0 (0/6) | 0 (0/18) | 0 (0/8) | － | 0 (0/1) | 0 (0/1) | － | 0 (0/44) |
| Hoping int'l seaport | － | 0 (0/6) | 0 (0/11) | 0 (0/8) | 0 (0/3) | 0 (0/3) | 0 (0/3) | － | － | － | － | － | － | 0 (0/34) |
| Hualien int'l airport | － | － | － | － | － | － | － | － | － | － | － | － | 0 (0/1) | 0 (0/1) |
| Hualien int'l seaport | 0 (0/1) | － | 0 (0/8) | 8.33% (1/12) | 0 (0/8) | 0 (0/7) | 0 (0/6) | － | 0 (0/5) | － | 0 (0/4) | 0 (0/1) | 0 (0/6) | 1.72% (1/58) |
| Kaohsiung int'l airport | － | 0 (0/1) | 0 (0/3) | － | 0 (0/2) | 0 (0/1) | － | － | － | － | － | － | － | 0 (0/7) |
| Kaohsiung int'l seaport | － | － | 25.00% (1/4) | 0 (0/2) | － | 0 (0/2) | 33.33% (2/6) | 9.09% (1/11) | 50.00% (3/6) | 50.00% (2/4) | 0 (0/2) | 33.33% (1/3) | 0 (0/4) | 22.73% (10/44) |
| Keelung int'l seaport | － | － | － | － | 0 (0/1) | － | － | 0 (0/7) | － | － | － | － | 0 (0/1) | 0 (0/9) |
| Liaoluo int'l seaport | 0 (0/17) | 0 (0/20) | 0 (0/7) | 0 (0/4) | － | － | － | 0 (0/2) | － | － | － | － | － | 0 (0/50) |
| Magong int'l seaport | － | － | － | － | 0 (0/1) | － | － | － | － | － | － | － | 0 (0/1) | 0 (0/2) |
| Mailiao int'l seaport | － | － | 66.67% (2/3) | － | 0 (0/2) | － | － | － | － | － | － | 0 (0/1) | － | 33.33% (2/6) |
| Other nameless/small ports in Matsu | － | － | － | 0 (0/1) | － | － | － | － | － | － | － | － | － | 0 (0/1) |
| Shuitou int'l seaport | 14.81% (4/27) | 4.65% (2/43) | 0 (0/6) | 0 (0/11) | － | － | － | 0 (0/3) | － | － | － | － | － | 6.67% (6/90) |
| Suao int'l seaport | － | － | － | － | － | － | － | － | － | － | － | 0 (0/1) | 0 (0/2) | 0 (0/3) |
| Taichung int'l seaport | 0 (0/1) | － | － | 0 (0/1) | － | － | － | － | － | － | － | 0 (0/1) | 0 (0/1) | 0 (0/4) |
| Taipei int'l airport | － | － | － | － | 0 (0/4) | － | － | 0 (0/2) | 0 (0/4) | 0 (0/1) | － | － | － | 0 (0/8) |
| Taipei int'l seaport | － | － | － | － | 0 (0/1) | － | － | － | － | － | － | － | － | 0 (0/1) |
| Taoyuan int'l airport | 8.96% (6/67) | 0 (0/29) | 0 (0/32) | 0 (0/12) | 0 (0/5) | 0 (0/7) | 14.29% (1/7) | 0 (0/21) | 0 (0/2) | － | 0 (0/5) | 0 (0/5) | 0 (0/4) | 3.57% (7/196) |
| Xinhu/Fuguodun/Lotsuo fishing ports | － | 0 (0/3) | － | － | － | － | － | － | － | － | － | － | － | 0 (0/3) |
| Subtotal | 8.85% (10/113) | 1.96% (2/102) | 3.95% (3/76) | 1.92% (1/52) | 0 (0/30) | 0 (0/24) | 10.71% (3/28) | 1.56% (1/64) | 13.64% (3/22) | 40.00% (2/5) | 0 (0/12) | 7.69% (1/13) | 0 (0/20) | 4.63% (26/561) |
| **(E) Total** | 14.26% (69/484) | 10.68% (61/571) | 7.84% (44/561) | 7.49% (51/681) | 8.95% (88/983) | 6.13% (50/815) | 10.4% (67/644) | 7.6% (50/658) | 10.27% (23/224) | 6.84% (21/307) | 1.74% (5/287) | 6.44% (19/295) | 4.20% (12/286) | 8.24% (560/6,796) |
