# Supplementary material for: Delayed correlation between the incidence rate of indigenous murine typhus in humans and the seropositive rate of Rickettsia typhi infection in small mammals in Taiwan from 2007–2019
Source: PLoS Negl Trop Dis. 2022 Apr 25;16(4):e0010394. doi: 10.1371/journal.pntd.0010394 (PMC9071160; doi:10.1371/journal.pntd.0010394)
Supplement: S3 Table — (DOCX) [file pntd.0010394.s003.docx]

**S3 Table.** Incidence rate (per 100,000 person-years) of indigenous murine typhus for each administrative district from 2007–2019.

| **District** | **2007** | **2008** | **2009** | **2010** | **2011** | **2012** | **2013** | **2014** | **2015** | **2016** | **2017** | **2018** | **2019** | **Total (2007-2019)** |
| --- | --- | --- | --- | --- | --- | --- | --- | --- | --- | --- | --- | --- | --- | --- |
| **Taichung** | 0.12 (3/2,596,274 *100,000) | 0.11 (3/2,615,690 *100,000) | 0.11 (3/2,629,323 *100,000) | 0.11 (3/2,639,905 *100,000) | 0.15 (4/2,655,456 *100,000) | 0.04 (1/2,674,359 *100,000) | 0.07 (2/2,693,892 *100,000) | 0.07 (2/2,708,338 *100,000) | 0(0/2,731,500 *100,000) | 0(0/2,754,191 *100,000) | 0(0/2,776,579 *100,000) | 0.04 (1/2,794,933 *100,000) | 0.14 (4/2,810,219 *100,000) | 0.07 (26/35,080,659 *100,000) |
| **Taipei** | 0(0/2,627,990 *100,000) | 0.04 (1/2,630,191 *100,000) | 0.04 (1/2,616,520 *100,000) | 0(0/2,602,868 *100,000) | 0(0/2,635,766 *100,000) | 0(0/2,663,263 *100,000) | 0(0/2,681,554 *100,000) | 0(0/2,693,672 *100,000) | 0.07 (2/2,706,030 *100,000) | 0(0/2,702,925 *100,000) | 0(0/2,689,845 *100,000) | 0(0/2,675,713 *100,000) | 0(0/2,657,652 *100,000) | 0.01 (4/34,583,989 *100,000) |
| **Taitung** | 0(0/234,672 *100,000) | 0(0/232,663 *100,000) | 0(0/232,071 *100,000) | 0(0/231,737 *100,000) | 0.44 (1/229,420 *100,000) | 0.44 (1/227,194 *100,000) | 0(0/225,407 *100,000) | 0(0/225,041 *100,000) | 0(0/223,189 *100,000) | 0(0/221,504 *100,000) | 0(0/220,083 *100,000) | 0.46 (1/219,643 *100,000) | 0(0/217,721 *100,000) | 0.10 (3/2,940,345 *100,000) |
| **Tainan** | 0.05 (1/1,868,001 *100,000) | 0.05 (1/1,871,509 *100,000) | 0.16 (3/1,873,681 *100,000) | 0.11 (2/1,873,571 *100,000) | 0.21 (4/1,874,724 *100,000) | 0.21 (4/1,878,795 *100,000) | 0.27 (5/1,882,526 *100,000) | 0.16 (3/1,883,042 *100,000) | 0.27 (5/1,885,376 *100,000) | 0.16 (3/1,885,388 *100,000) | 0.48 (9/1,886,502 *100,000) | 0.05 (1/1,884,717 *100,000) | 0.11 (2/1,882,313 *100,000) | 0.18 (43/24,430,145 *100,000) |
| **Hualian** | 0(0/344,087 *100,000) | 0(0/342,516 *100,000) | 0(0/340,903 *100,000) | 0(0/339,671 *100,000) | 0(0/337,557 *100,000) | 0(0/336,020 *100,000) | 0(0/334,468 *100,000) | 0(0/333,630 *100,000) | 0.30 (1/332,424 *100,000) | 0(0/331,372 *100,000) | 0(0/329,976 *100,000) | 0(0/328,749 *100,000) | 0(0/326,986 *100,000) | 0.02 (10/4,358,359 *100,000) |
| **Kinmen** | 0(0/79,023 *100,000) | 1.20 (1/83,225 *100,000) | 0(0/89,471 *100,000) | 0(0/95,145 *100,000) | 0(0/99,691 *100,000) | 0(0/108,147 *100,000) | 0(0/116,570 *100,000) | 0(0/123,947 *100,000) | 0(0/129,831 *100,000) | 0(0/133,185 *100,000) | 0(0/135,563 *100,000) | 0(0/137,837 *100,000) | 0(0/139,281 *100,000) | 0.07 (10/1,470,916 *100,000) |
| **Nantou** | 0(0/533,903 *100,000) | 0(0/532,762 *100,000) | 0.19 (1/531,307 *100,000) | 0(0/528,283 *100,000) | 0(0/524,426 *100,000) | 0.19 (1/521,218 *100,000) | 0(0/518,738 *100,000) | 0(0/515,784 *100,000) | 0(0/511,518 *100,000) | 0(0/507,210 *100,000) | 0(0/503,261 *100,000) | 0(0/499,194 *100,000) | 0.20 (1/495,283 *100,000) | 0.04 (3/6,722,887 *100,000) |
| **Pingtung** | 0.90 (8/890,753 *100,000) | 0.68 (6/886,786 *100,000) | 0.45 (4/883,075 *100,000) | 0.80 (7/878,062 *100,000) | 0.46 (4/868,827 *100,000) | 0.58 (5/861,373 *100,000) | 0.35 (3/855,345 *100,000) | 0.59 (5/850,527 *100,000) | 1.07 (9/843,981 *100,000) | 0(0/838,584 *100,000) | 0.96 (8/832,489 *100,000) | 0.36 (3/828,275 *100,000) | 0.73 (6/821,834 *100,000) | 0.61 (68/11,139,911 *100,000) |
| **Miaoli** | 0.18 (1/559,776 *100,000) | 0(0/559,851 *100,000) | 0(0/560,581 *100,000) | 0.18 (1/559,921 *100,000) | 0(0/560,813 *100,000) | 0.18 (1/562,684 *100,000) | 0(0/564,647 *100,000) | 0(0/566,446 *100,000) | 0(0/565,704 *100,000) | 0(0/561,495 *100,000) | 0(0/556,324 *100,000) | 0(0/551,082 *100,000) | 0(0/546,690 *100,000) | 0.04 (3/7,276,014 *100,000) |
| **Taoyuan** | 0.16 (3/1,921,526 *100,000) | 0.05 (1/1,945,581 *100,000) | 0.05 (1/1,967,038 *100,000) | 0.05 (1/2,017,047 *100,000) | 0.05 (1/2,005,795 *100,000) | 0(0/2,020,103 *100,000) | 0(0/2,036,708 *100,000) | 0(0/2,049,009 *100,000) | 0(0/2,086,081 *100,000) | 0.05 (1/2,124,339 *100,000) | 0.05 (1/2,167,616 *100,000) | 0(0/2,204,824 *100,000) | 0(0/2,235,005 *100,000) | 0.03 (9/26,780,672 *100,000) |
| **Kaohsiung** | 0.58 (16/2,760,397 *100,000) | 0.33 (9/2,766,343 *100,000) | 0.40 (11/2,769,072 *100,000) | 0.58 (16/2,770,682 *100,000) | 0.22 (6/2,772,461 *100,000) | 0.36 (10/2,775,482 *100,000) | 0.50 (14/2,779,092 *100,000) | 0.22 (6/2,777,296 *100,000) | 0.25 (7/2,778,835 *100,000) | 0.18 (5/2,778,092 *100,000) | 0.36 (10/2,778,023 *100,000) | 0.36 (10/2,773,932 *100,000) | 0.25 (7/2,773,177 *100,000) | 0.35 (127/36,052,884 *100,000) |
| **Yunlin** | 0(0/726,868 ) | 0.28 (2/725,237 ) | 0.14 (1/723,065 ) | 0.14 (1/718,671 ) | 0(0/715,288 ) | 0.28 (2/711,887 ) | 0.14 (1/709,491 ) | 0(0/707,282 ) | 0(0/701,898 ) | 0.29 (0/697,105 ) | 0.29 (2/692,570 ) | 0(0/688,717 ) | 0.15 (1/683,318 ) | 0.13 (12/9,201,397 ) |
| **New Taipei** | 0.11 (4/3,779,219 *100,000) | 0.05 (2/3,811,043 *100,000) | 0.05 (2/3,849,492 *100,000) | 0.08 (3/3,880,743 *100,000) | 0(0/3,903,745 *100,000) | 0.03 (1/3,926,188 *100,000) | 0(0/3,945,789 *100,000) | 0(0/3,956,728 *100,000) | 0.03 (1/3,966,052 *100,000) | 0(0/3,972,204 *100,000) | 0.03 (1/3,982,434 *100,000) | 0.03 (1/3,987,520 *100,000) | 0.05 (2/4,004,598 *100,000) | 0.03 (17/50,965,755 *100,000) |
| **Hsinchu City** | 0.25 (1/396,983 *100,000) | 0(0/402,368 *100,000) | 0(0/408,466 *100,000) | 0(0/412,893 *100,000) | 0(0/417,335 *100,000) | 0(0/422,554 *100,000) | 0(0/427,050 *100,000) | 0(0/430,041 *100,000) | 0(0/432,860 *100,000) | 0(0/435,238 *100,000) | 0(0/439,299 *100,000) | 0(0/443,351 *100,000) | 0(0/447,359 *100,000) | 0.02 (1/5,515,797 *100,000) |
| **Hsinchu County** | 0.20 (1/491,405 *100,000) | 0(0/499,372 *100,000) | 0(0/506,546 *100,000) | 0(0/509,802 *100,000) | 0(0/515,044 *100,000) | 0(0/520,234 *100,000) | 0(0/526,920 *100,000) | 0(0/533,851 *100,000) | 0(0/539,173 *100,000) | 0(0/544,624 *100,000) | 0(0/549,580 *100,000) | 0(0/554,541 *100,000) | 0(0/559,734 *100,000) | 0.01 (10/6,850,826 *100,000) |
| **Chiayi City** | 0(0/272,718 *100,000) | 0(0/273,789 *100,000) | 0(0/274,171 *100,000) | 0(0/272,882 *100,000) | 0.37 (1/272,128 *100,000) | 0(0/271,689 *100,000) | 0(0/271,291 *100,000) | 0(0/270,841 *100,000) | 0(0/270,896 *100,000) | 0(0/270,254 *100,000) | 0(0/269,717 *100,000) | 0(0/268,951 *100,000) | 0(0/268,435 *100,000) | 0.03 (1/3,527,762 *100,000) |
| **Chiayi County** | 0(0/551,993 *100,000) | 0(0/549,265 *100,000) | 0(0/547,550 *100,000) | 0(0/544,782 *100,000) | 0.19 (1/540,059 *100,000) | 0(0/535,045 *100,000) | 0(0/531,026 *100,000) | 0(0/526,936 *100,000) | 0.19 (1/521,591 *100,000) | 0(0/517,339 *100,000) | 0(0/513,082 *100,000) | 0(0/509,161 *100,000) | 0(0/504,750 *100,000) | 0.03 (2/6,892,579 *100,000) |
| **Changhua** | 0.46 (6/1,313,986 *100,000) | 0.08 (1/1,313,003 *100,000) | 0.76 (10/1,312,165 *100,000) | 0.31 (4/1,308,926 *100,000) | 0.23 (3/1,304,216 *100,000) | 0.46 (6/1,300,618 *100,000) | 0.08 (1/1,297,968 *100,000) | 0.23 (3/1,293,530 *100,000) | 0.62 (8/1,289,274 *100,000) | 0.16 (2/1,288,803 *100,000) | 0.47 (6/1,284,423 *100,000) | 0.31 (4/1,279,276 *100,000) | 0.31 (4/1,274,724 *100,000) | 0.34 (58/16,860,912 *100,000) |
| **Yilan** | 0(0/460,133 *100,000) | 0(0/461,082 *100,000) | 0(0/461,461 *100,000) | 0(0/460,604 *100,000) | 0(0/459,349 *100,000) | 0(0/458,616 *100,000) | 0(0/458,602 *100,000) | 0(0/458,771 *100,000) | 0(0/458,313 *100,000) | 0(0/457,808 *100,000) | 0(0/457,106 *100,000) | 0(0/456,006 *100,000) | 0(0/454,706 *100,000) | 0(0/5,962,557 *100,000) |
| **Keelung** | 0(0/390,299 *100,000) | 0(0/389,443 *100,000) | 0(0/388,703 *100,000) | 0(0/385,869 *100,000) | 0(0/381,770 *100,000) | 0(0/378,301 *100,000) | 0(0/376,082 *100,000) | 0(0/373,786 *100,000) | 0(0/372,787 *100,000) | 0(0/371,837 *100,000) | 0(0/371,876 *100,000) | 0(0/370,795 *100,000) | 0(0/369,475 *100,000) | 0(0/4,921,023 *100,000) |
| **Penghu** | 0(0/92,077 *100,000) | 0(0/92,602 *100,000) | 0(0/94,886 *100,000) | 0(0/96,388 *100,000) | 0(0/96,597 *100,000) | 0(0/97,724 *100,000) | 0(0/99,276 *100,000) | 0(0/101,112 *100,000) | 0(0/101,753 *100,000) | 0(0/102,329 *100,000) | 0(0/103,406 *100,000) | 0(0/104,013 *100,000) | 0(0/104,676 *100,000) | 0(0/1,286,839 *100,000) |
| **Matsu** | 0(0/9,814 *100,000) | 0(0/9,941 *100,000) | 0(0/9,798 *100,000) | 0(0/9,929 *100,000) | 0(0/10,010 *100,000) | 0(0/10,253 *100,000) | 0(0/11,771 *100,000) | 0(0/12,426 *100,000) | 0(0/12,496 *100,000) | 0(0/12,536 *100,000) | 0(0/12,716 *100,000) | 0(0/13,044 *100,000) | 0(0/13,095 *100,000) | 0(0/147,829 *100,000) |
| **Nationwide** | 0.19 (44/22,635,162 *100,000) | 0.12 (27/22,730,107 *100,000) | 0.16 (37/22,807,741 *100,000) | 0.17 (38/22,880,314 *100,000) | 0.11 (25/22,924,538 *100,000) | 0.14 (32/23,008,476 *100,000) | 0.11 (26/23,093,324 *100,000) | 0.08 (19/23,143,525 *100,000) | 0.15 (34/23,217,977 *100,000) | 0.06 (13/23,269,065 *100,000) | 0.16 (37/23,317,006 *100,000) | 0.09 (21/23,341,563 *100,000) | 0.12 (27/23,362,419 *100,000) | 0.13 (380/299,731,217 *100,000) |
