# Supplementary material for: Delayed correlation between the incidence rate of indigenous murine typhus in humans and the seropositive rate of Rickettsia typhi infection in small mammals in Taiwan from 2007–2019
Source: PLoS Negl Trop Dis. 2022 Apr 25;16(4):e0010394. doi: 10.1371/journal.pntd.0010394 (PMC9071160; doi:10.1371/journal.pntd.0010394)
Supplement: Attachment — Submitted filename: Comments.docx [file pntd.0010394.s004.docx]

**Title**: Delayed Correlation between the Incidence Rate of Indigenous Murine Typhus in Humans and the Seropositive Rate of Rickettsia typhi Infection in Small Mammals in Taiwan from 2007–2019

**Comments**:

1. It is to be noted that this study demonstrate only a moderate co-relation with occurrence of human cases corresponding to sero-positive rate in small mammals at 1 or 2 month prior. Given the low sample size [human cases (n=380 over 13 years) as well as sero-positive small mammals (only 8%)], it is difficult to interpret the effect of this co-relation in actual scenario. It is therefore suggested to highlight limitations of the present study. The term ‘moderately positive co-relation’ should be used for emphasizing the results.

2. Surveyed region wise as well as year wise positivity data on human and small mammals (indicating the rodent species too) should be presented, preferably in a table format.

3. Figure resolution needs to be increased.
