# Supplementary material for: Delayed correlation between the incidence rate of indigenous murine typhus in humans and the seropositive rate of Rickettsia typhi infection in small mammals in Taiwan from 2007–2019
Source: PLoS Negl Trop Dis. 2022 Apr 25;16(4):e0010394. doi: 10.1371/journal.pntd.0010394 (PMC9071160; doi:10.1371/journal.pntd.0010394)
Supplement: Attachment — Submitted filename: Response to reviewers v2.docx [file pntd.0010394.s005.docx]

Dear Editor,

We appreciate the time and efforts you and the reviewers have spent on reviewing our manuscript and providing valuable feedback. The reviewer’s comments have been carefully considered and answered. Please see below, for a point-by-point response to the reviewers’ comments and concerns.

We look forward to hearing from you. If you have any queries, please don’t hesitate to contact me.

Best regards.

Sincerely yours,

Dr. Hwa-Jen Teng

Corresponding author: Hwa-Jen Teng

E-mail: hjteng@cdc.gov.tw

**Response to reviewers**

**Reviewer #1**

**1-1.** It is to be noted that this study demonstrate only a moderate co-relation with occurrence of human cases corresponding to sero-positive rate in small mammals at 1 or 2 month prior. Given the low sample size [human cases (n=380 over 13 years) as well as sero-positive small mammals (only 8%)], it is difficult to interpret the effect of this co-relation in actual scenario. It is therefore suggested to highlight limitations of the present study. The term ‘moderately positive co-relation’ should be used for emphasizing the results.

**Reply:** Thanks for your comment. This comment was followed by restating the sentence in the section of Results and adding description of study limitations in the Discussion section as following:

(1) Page 17 line 268-271 (in the Results section): Although a synchronous correlation was not observed, the 1-month-delayed and 2-month-delayed incidence rates were both **moderately positive** correlated with the prior seropositive rate in small mammals.

(2) Page 26 line 386-389 (in the Discussion section): Given the moderately positive correlation and limited number of confirmed domestic patients observed in this study, the actual effect of the proposed early reminder system is still needed to clarify with further studies.

**1-2.** Surveyed region wise as well as year wise positivity data on human and small mammals (indicating the rodent species too) should be presented, preferably in a table format.

**Reply:** This comment was followed by further providing two supplementary tables. Seropositive rate in small mammals and incidence rate of indigenous murine typhus [including numerator (positivity or confirmed cases) and denominator (sample/population size)] for each surveyed region/year were additionally provided in S2 Table and S3 Table, respectively. The display of animal data was stratified by rodent species. The description of the provided supplementary data was restating in the sections of Materials and methods and Supporting information as following:

(1) Page 11 line 179-181 (in the Materials and methods section): The seropositive rate of *R. typhi* in small mammals, number of cases and incidence rate of indigenous murine typhus for each month/year/region/rodent species (animal data) are shown in the supplementary S1-3 Table.

(2) Page 38 line 621 & page 39 line 622-624 (in the Supporting information section): Captions of the further added S2 Table and S3 Table were listed.

**1-3.** Figure resolution needs to be increased.

**Reply:** Thank you for pointing this out. This comment was followed by increasing the resolution of each figure. The clarity of all figures had been further ensured by Preflight Analysis and Conversion Engine (PACE) digital diagnostic tool/software to meet *PLOS* requirements.

**Reviewer #2**

**2-1.** The correlation is established using monthly data from the entire Taiwan. Given the large variations of human infection incidents and rodent seropositive rates across Taiwan, it might be worthwhile to further investigate sub-regions, especially those having geographical overlapping of sampling sites.

**Reply:** We agree with you. Therefore, to realize whether the correlation observed in data from entire Taiwan is also presented in the limited region with high level of incidence rate in human and seropositive rate in small mammals, we had additionally analyzed the data from the overlapping region and presented in Table 2 of the original manuscript. To improve readability of this issue, this comment was reflected by restating one sentence in the section of Results as following:

Page 20 line 290-295: Furthermore, if the area was limited to places with high incidence rates of indigenous infection **(all the mentioned four ports with the highest seropositivity of anti-*R. typhi* responses are also inside/near these places)**, the regression model, which was constructed by the same variables, could **still** account for 16.16% and 18.23% of the incidence rate of indigenous human murine typhus in the subsequent one and two months, respectively (Table 2).

**2-2.** Choice of secondary antibody for IFA assay of animal serum included mixture of IgG/IgA/IgM. Since IgG has a much longer half life, using specific IgA and/or IgM secondary antibodies might better differentiate more recent infections versus infections that occurred months ago.

**Reply:** Thanks for providing this insight. The comment was followed by restating the sentence and adding description in the section of Discussion as following:

Page 29 lines 437-441: Surveillance on animals in this study was based on the detection of anti-*R. typhi* responses but not the pathogen. **Detected immunoglobulin types were not further differentiated.** **The proportion attributable to recent infections still needs to be further studied (e.g. using IgA and/or IgM-specific secondary antibodies)**.
